# Surface and interfacial sciences for future technologies

**DOI:** 10.1093/nsr/nwae272

**Published:** 2024-08-02

**Authors:** Xiong Zhou, Qian Shen, Yongfeng Wang, Yafei Dai, Yongjun Chen, Kai Wu

**Affiliations:** College of Chemistry and Molecular Engineering, Peking University, Beijing 100871, China; Department of Interdisciplinary Sciences, National Natural Science Foundation of China, Beijing 100085, China; School of Electronics, Peking University, Beijing 100871, China; Department of Interdisciplinary Sciences, National Natural Science Foundation of China, Beijing 100085, China; Department of Interdisciplinary Sciences, National Natural Science Foundation of China, Beijing 100085, China; College of Chemistry and Molecular Engineering, Peking University, Beijing 100871, China

**Keywords:** interfacial science, interfacial state, superconductivity, catalysis, device

## Abstract

Physical science has undergone an evolutional transition in research focus from solid bulks to surfaces, culminating in numerous prominent achievements. Currently, it is experiencing a new exploratory phase—interfacial science. Many a technology with a tremendous impact is closely associated with a functional interface which delineates the boundary between disparate materials or phases, evokes complexities that surpass its pristine comprising surfaces, and thereby unveils a plethora of distinctive properties. Such an interface may generate completely new or significantly enhanced properties. These specific properties are closely related to the interfacial states formed at the interfaces. Therefore, establishing a quantitative relationship between the interfacial states and their functionalities has become a key scientific issue in interfacial science. However, interfacial science also faces several challenges such as invisibility in characterization, inaccuracy in calculation, and difficulty in precise construction. To tackle these challenges, people must develop new strategies for precise detection, accurate computation, and meticulous construction of functional interfaces. Such strategies are anticipated to provide a comprehensive toolbox tailored for future interfacial science explorations and thereby lay a solid scientific foundation for several key future technologies.

## Teaser

This review concisely describes the key roles of surface and interfacial sciences in developments of several cutting-edge technologies in the future.

## INTRODUCTION

**Figure 1.**
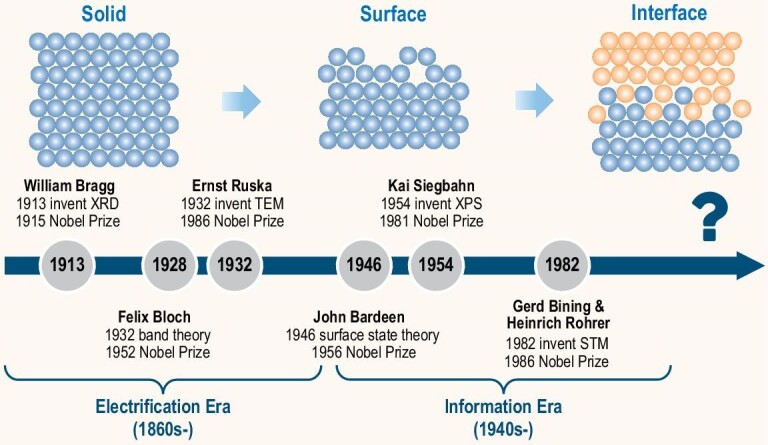
Established and projected evolutions of physical science from bulk solids to surfaces to interfaces.

Since the invention of electric lights in the 19th century, humanity has technologically entered the era of electrification. In this era there appeared a series of solid state detection technologies (Fig. 1) one after another, including X-ray diffraction for detection of solid lattice structures (invented by Bragg in 1913), transmission electron microscopy for visualization of solid structures (invented by Ruska in 1932), and solid state nuclear magnetic resonance technology for determination of chemical bonds and molecular structures (invented by Bloch and Purcell in 1946). Concurrently, solid state theory also experienced significant breakthroughs like Bloch's band theory proposed in 1928 to provide a fundamental framework for understanding the conductivity of solids such as metals, insulators, and semiconductors. The emergence of these solid state technologies and theories greatly pushed the rapid development of electronic technologies. Initially, understanding of solids was primarily focused on bulk crystals of periodic structures with theoretical descriptions of the atoms within their periodic unit cells. However, solid surfaces routinely exhibit non-periodic and discontinuous characteristics which remarkably increase the difficulties of theoretical descriptions and experimental characterizations, thereby posing new challenges to the scientific community of the time. World-renowned physicist Pauli once stated: ‘God made solids, but surfaces were the work of the devil!’ To address this challenge, the surface science community had devoted itself to the development of various surface characterization techniques such as X-ray photoelectron spectroscopy for surface spectroscopic analysis (invented by Siegbahn in 1954) and scanning tunneling microscopy for surface atomic imaging (invented by Binnig and Rohrer in 1982) [1]. In terms of surface theory, Bardeen proposed the theory of semiconductor surface states in 1946, which laid the groundwork for the invention of the transistor in 1947 and marked that mankind had stepped into the information era.

With the rapid progress of modern techniques, physical science has entered a new exploratory phase, interfacial science. An interface, being the boundary between two distinct materials or phases, is much more complex than its constituent surfaces. Breakthroughs in both technical and theoretical aspects of interfaces are on the cusp of ushering in a transformative leap within the realm of physical science. It has been appreciated that numerous techniques with prominent impacts are closely associated with interfaces. For instance, the transistor, a core elementary unit in chip technology, has been highlighted by Kroemer [2] with the assertion, ‘the interface is the device’. Similarly, interface superconductivity is anticipated to speed up revolutionary advances in superconductors. Many important industrial chemical processes heavily rely on supported heterogeneous catalysts where their performance optimizations critically depend on the metal-support interfaces. Highly efficient quantum computations mainly lie in the Josephson junction which is essentially a functional interface between a superconductor and an insulator. In the realm of sensors, the conversion of external information into electrical signals is primarily achieved at the interfaces. Obviously, the pursuit of in-depth explorations in interfacial science has evolved as a pivotal driving force for the advancements of the described key technologies for the future. Anticipated extension and reference of existing techniques and theories in surface science into interfacial science, however, face a formidable challenge that physical science must address, particularly in the advancement of low-dimensional and functional interfacial systems.

## THE IMPORTANCE OF INTERFACES

### Interfaces in superconductivity

In 1911, the phenomenon of superconductivity was first noticed and disclosed by Onnes who observed that the resistance of mercury abruptly disappeared as the temperature was cooled down to 4 K. Following this discovery, extensive research had unfolded and lasted for decades until the landmark superconducting quantum theory, now known as BCS theory, was introduced in 1957 by Bardeen, Cooper, and Schrieffer [3]. These scientists eventually elucidated that in the superconducting metals, electrons can attract one another to form pairs through the mediation of lattice vibrations. These paired electrons move in unison to generate the superconducting current. Their disruption requires a specific energy surpassing a threshold, which leads to the occurrence of an energy gap between the ground and excited states of the superconductor, termed the superconducting energy gap. The discovery of a high-temperature superconductor system consisting of barium lanthanum copper oxide by Bednorz and Müller [4,5] in 1986 had significantly increased the superconducting temperature up to 30 K and sparked an intensified search for high-temperature superconductors. It's been a dream for scientists to achieve superconductivity at the temperature of liquid nitrogen (77 K) at which large-scale industrial applications would come true, setting the stage for various technological breakthroughs. Currently, the highest transition temperature of copper-based high-temperature superconductors under ambient pressure is reported to be 133.5 K [6]. Despite these advancements, room-temperature superconductivity remains an elusive goal.

**Figure 2.**
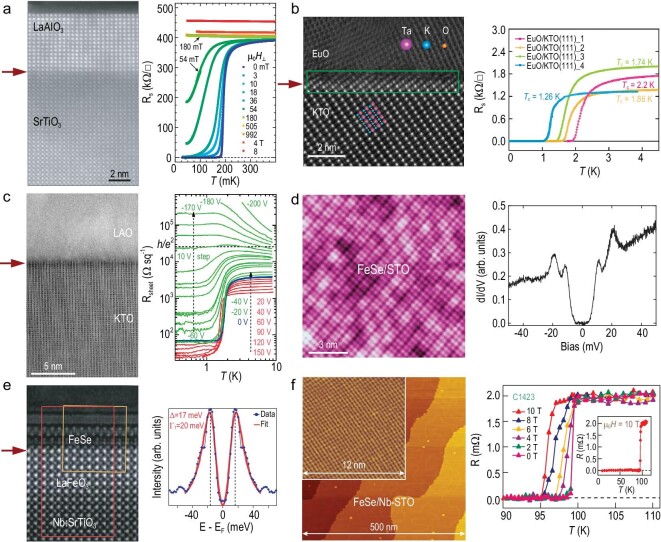
Several investigated interface systems in superconductivity, with interfaces marked by red arrows. (a) The LaAlO_3_/SrTiO_3_ interface possesses a superconducting transition temperature of ∼200 mK [8]. Copyright 2007, American Association for the Advancement of Science. (b) The EuO/KTO interface exhibits superconductivity with a transition temperature of ∼2.2 K [9]. Copyright 2021, American Association for the Advancement of Science. (c) A superconducting state is evoked at the LaAlO_3_/KTaO_3_ interface under the influence of an electric field [10]. Copyright 2021, American Association for the Advancement of Science. (d) Superconductivity is identified at the interface of a FeSe monolayer and its underlying SrTiO_3_ substrate [11]. Copyright 2012, IOP science. (e) The superconducting transition temperature at the interface of a FeSe thin film and LaFeO_3_ is close to 80 K [15]. Copyright 2021, Springer Nature. (f) The superconducting transition temperature at the interface of a FeSe monolayer and doped SrTiO_3_ substrate is ∼100 K [17]. Copyright 2015, Springer Nature.

The ongoing efforts to raise the transition temperature of high-temperature superconductors have eventually focused on two research avenues. One involves the exploration of superconductivity under high pressure, while the other, as predicted by Ginzburg [7], suggests that ‘a two-dimensional (surface) superconductivity is also conceivable’. In 2007, Mannhart *et al.* [8] made a pivotal discovery that interfaces between two insulating oxides (LaAlO_3_/SrTiO_3_) exhibit superconducting properties (Fig. 2a), marking a novel direction in superconductivity research which is now known as interface superconductivity. Following this, Bhattacharya *et al.* [9] leveraged interfaces between KTaO_3_ and EuO (or LaAlO_3_) to increase the superconducting temperature to 2.2 K (Fig. 2b), significantly surpassing that for the LaAlO_3_/SrTiO_3_ system, i.e. 200 mK. Additionally, research by Xie [10] demonstrated the induction of a quantum phase transition between superconducting and insulating states at the LaAlO_3_/KTaO_3_ interface via the application of an electric field (Fig. 2c).

The advancements in superconductivity observed in FeSe monolayers grown on oxide substrates demonstrate a remarkable enhancement over bulk FeSe, opening new possibilities for high-temperature superconductivity research and applications. In 2012, Xue *et al.* [11–14] reported a superconducting gap of ∼20 meV in monolayered FeSe grown on the SrTiO_3_ substrate, which is an eightfold increase compared to bulk FeSe. This significant increase is attributed to the interface effects between the FeSe monolayer and the SrTiO_3_ substrate (Fig. 2d). Based on this discovery, Feng *et al.* [15,16] elevated the superconducting transition temperature (Tc) from 46 K to nearly 80 K by modifying the oxide substrates used for growth of the FeSe films. These substrates included SrVO_3_ and LaFeO_3_, showcasing the potential of substrate engineering to enhance superconductivity in these systems (Fig. 2e). Furthermore, in 2015, Jia *et al.* [17] detected superconducting signals at an even higher temperature of ∼100 K in monolayered FeSe films grown on the Nb-doped SrTiO_3_ substrate (Fig. 2f).

These interface superconductor systems not only hold immense promise for pushing the boundaries of high-temperature superconductivity but also offer a critical platform for exploring the underlying mechanisms driving these phenomena. As research continues, breakthroughs in interface superconductivity are expected to have significant implications across various technological fields, including nuclear fusion, magnetic levitation, and power transmission. The study of these interfaces is poised to unlock new pathways and advancements, potentially revolutionizing how superconductivity is understood and applied.

### Interfaces in catalysis

The concept of catalysis, introduced by Berzelius in 1836, has a rich and storied history. Initially, the development of catalysts was largely empirical, relying on trial-and-error methods. This approach limited the mechanistic understanding of catalytic processes, effectively treating catalysts as ‘black boxes.’ Catalysts are generally divided into two main categories: homogeneous and heterogeneous. Heterogeneous catalysts are critical in industrial processes that dominate the energy and chemical sectors. The crucial role of surfaces in heterogeneous catalysis was not fully appreciated until the early 20th century, when Langmuir systematically applied surface chemistry techniques to the study of catalysts. His work laid the groundwork for understanding how reactions occur on catalyst surfaces. In 1925, active site theory developed by Taylor *et al.* [18,19] further advanced this understanding by demonstrating that catalytic activity is concentrated at specific sites on the catalyst surface, rather than being uniformly distributed across the entire catalyst. This theory has since become a cornerstone of modern catalysis, providing a more nuanced and detailed understanding of how catalysts function at the molecular level.

The progress of catalysis research has increasingly underscored the significance of interfacial phenomena in catalytic processes. The research on ammonia synthesis by Ertl [20] demonstrated how the Al₂O₃-Fe interface can alter the structural configuration of iron, shifting it from a less active crystalline face to the more reactive (111) or (211) planes. This modification is crucial for the efficient activation of nitrogen molecules, which are typically inert. In 1978, Tauster *et al.* [21,22] introduced the concept of strong metal-support interaction (SMSI), highlighting the profound impact that the metal-support interface has on the catalytic performance of metals. SMSI emphasizes that the interaction between the metal and its support can significantly influence the properties and activity of the catalyst. Advancements in electrocatalysis throughout the 20th century have further emphasized the importance of the liquid-solid interface between the electrolyte and the solid electrode. This interface is critical in determining the efficiency of electrocatalytic processes, as it directly affects the reactions occurring at the electrode surface. These insights into interfacial phenomena continue to drive innovations in catalyst design and application, leading to more efficient and effective catalytic systems [23,24].

Towards the end of the previous century, there was a notable surge in research interest in Au catalysis, particularly following the discovery by Goodman *et al.* [25] in 1998. They found that the Au-TiO_2_ interface profoundly influences the catalytic properties of gold. This change is attributed to charge transfer to the Au surface via the TiO_2_-Au interface, resulting in a negative charge on the Au surface and significantly enhancing its CO oxidation activity. Subsequent investigations, such as those conducted by Bao *et al.* [26] in 2010, capitalized on interfacial confinement effects arising from strong interactions between precious metal surfaces and iron atoms in monolayered iron oxide films. They utilized this phenomenon for catalytic reactions, including the removal of trace CO in hydrogen environments. Moreover, dynamic confinement [27] and single atoms [28] were scrutinized under operating conditions to illustrate the dynamic behaviors of interface atoms. In 2011, Yang *et al.* [29] identified two distinct metal-metal oxide interfaces, CeO_2_-Pt and Pt-SiO_2_, which facilitate two sequential catalytic reactions. The CeO_2_-Pt interface catalyzed the decomposition of methanol into CO and H_2_, while the Pt-SiO_2_ interface facilitated ethylene hydroformylation. Expanding further on the significance of interfaces in catalysis, Murray *et al.* [30] demonstrated in 2013 that the CO oxidation is significantly enhanced at the ceria-metal interface sites across various group VIII metal catalysts, emphasizing the critical role of interfaces in catalytic processes. In 2014, Zheng *et al.* [31] utilized wet chemical techniques to create model nanocatalysts, enabling the investigation of precious metal-oxide interfacial effects. They evolved the reactive interfaces from conventional one-dimensional (1D) to three-dimensional structures, enabling various catalytic processes such as CO oxidation at ambient temperatures, selective oxidation of CO in hydrogen-rich conditions, and removal of trace hydrogen in oxygen-rich atmospheres.

**Figure 3.**
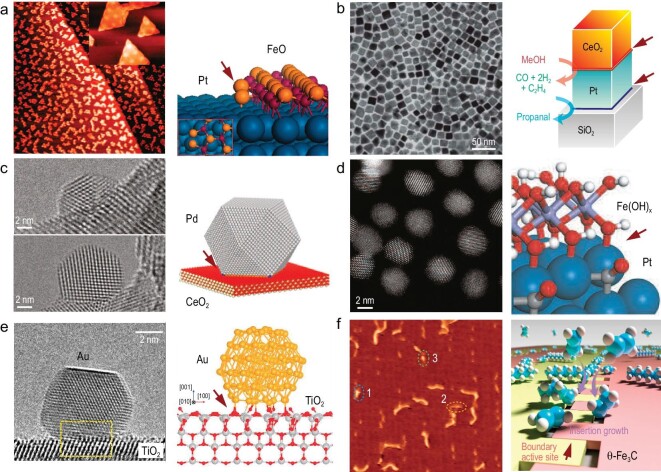
Several investigated interface systems in catalysis, with interfaces marked by red arrows. (a) Pt/FeO interface: utilized for the catalytic removal of trace CO in hydrogen streams [26]. This interface enhances the efficiency of CO oxidation, making it an effective catalyst for purifying hydrogen gas streams. Copyright 2010, American Association for the Advancement of Science. (b) CeO_2_–Pt and Pt–SiO_2_ interfaces: these distinct metal-metal oxide interfaces facilitate two separate tandem catalytic reactions, demonstrating the versatility and efficacy of different metal-oxide combinations in catalytic processes [29]. Copyright 2011, Springer Nature. (c) Ceria-metal interface: carbon monoxide oxidation is significantly enhanced at the ceria-metal interface, underscoring the importance of the metal-oxide boundary in catalytic activity [30]. Copyright 2013, American Association for the Advancement of Science. (d) Noble metal-oxide interfaces: the impact of these interfaces is showcased through nanocatalysts, which exhibit improved catalytic properties due to the interactions at the metal-oxide boundaries [31]. Copyright 2014, American Association for the Advancement of Science. (e) Au/TiO_2_ interface: epitaxial rotation at this interface enhances CO oxidation activity, highlighting the role of interface structure in determining catalytic performance [33]. Copyright 2021, American Association for the Advancement of Science. (f) Iron carbide domains: surface polymerization of ethylene is facilitated at the interface of iron carbide domains, demonstrating the role of specific interface configurations in promoting catalytic reactions [35]. Copyright 2022, American Association for the Advancement of Science.

In 2017, Ma *et al.* [32] introduced a bifunctional carbide-supported gold catalyst, Au/α-MoC, which demonstrates remarkable performance in the water-gas shift reaction. This catalyst leveraged the low-temperature activation and dissociation of H_2_O by the cubic phase α-MoC, combined with the enhancement of CO adsorption and activation by dispersed gold. As a result, the water-gas shift reaction temperature was significantly reduced to 120°C, showcasing the catalytic potential of this novel interface. In 2021, Zhang *et al.* [33] emphasized the profound influence of the atomic structure of the Au/TiO_2_ interface and the rotation of gold nanoparticles on the TiO_2_ surface on CO oxidation activity. Their work underscored the importance of understanding the atomic-level interactions at interfaces for optimizing catalytic performance (Fig. 3e). Theoretical frameworks for the systematic design and selection of stable, anti-sintering nanocatalysts have been further developed by Li *et al.* [34]. They proposed a theory on the regulation of nanocatalyst growth kinetics through interfacial action and introduced a dual-function carrier high-throughput screening strategy, facilitating the discovery of advanced nanocatalysts with enhanced stability and activity. In 2022, Wu *et al.* [35] made a significant discovery regarding the interface of iron carbide domains acting as the active site for ethylene polymerization. This observation, occurring *in situ* at room temperature, highlights the critical role of catalyst interfaces in driving catalytic reactions and enhancing catalytic performance (Fig. 3f). These advancements underscore the importance of interface engineering in catalysis and highlight the potential for further innovations in catalyst design and application through a deeper understanding of interfacial phenomena.

The conversion and utilization of new energy sources heavily rely on heterogeneous catalysts, particularly those composed of metal catalysts supported on oxides. At the heart of these catalysts, metal atoms situated at the metal-oxide interfaces play a pivotal role as active centers for catalytic reactions [36–39]. The activity and selectivity of these catalysts are profoundly influenced by various factors, including the electronic characteristics, geometric configurations, and interactions of metal atoms with adjacent oxides. A deep understanding of the interactions at metal-oxide interfaces is essential for the design of highly efficient catalysts and the reduction of manufacturing costs. This knowledge holds significant implications for various aspects of energy and environmental sustainability, including China's new energy development initiatives, the global transition towards cleaner energy sources, efforts to mitigate climate change, and the pursuit of carbon peak and carbon neutrality objectives. By advancing our understanding of metal-oxide interface interactions and leveraging this knowledge in catalyst design, we can accelerate progress towards a more sustainable and environmentally benign energy landscape.

### Interfaces in devices

The creation of the PN junction by Ohl in 1940 marked a significant milestone in the history of electronics, ushering in a new era where the development of electronic devices became intricately linked with the study of interfaces. This junction, showcasing unidirectional conductivity between P- and N-type semiconductors, exemplifies the intimate relationship between device functionality and interfaces. Subsequent invention of transistor in 1947 by Shockley, Bardeen, and Brattain capitalized on the unique properties of interfaces to amplify and switch electrical signals, laying the groundwork for Noyce's groundbreaking invention of the first integrated circuit in 1961. This highlighted the indispensable role of transistors and, by extension, interfaces, in propelling the advancement of semiconductor technology. Today, modern information technology is built upon the foundation of chips, or large-scale integrated circuits, which have become deeply ingrained in various aspects of our daily life. At the core of these chips lie transistors which are essentially composed of multiple interfaces, including PN junctions (homogeneous semiconductor interfaces), heterojunctions (heterogeneous semiconductor interfaces), metal-semiconductor junctions, and MOS interfaces (encompassing metal-oxide-semiconductor interfaces, including oxide-semiconductor, metal-oxide, and oxide-oxide composite interfaces). These interfaces play a crucial role in determining the performance and functionality of electronic devices, enabling the miniaturization, efficiency, and versatility that characterize modern semiconductor technology. Thus, continuous exploration and understanding of interfaces remain paramount for driving innovation and progress in the field of electronics.

A crucial metric for assessing device performance is the interfacial trapped charge density at the semiconductor-oxide interface, commonly referred to as ‘interface states’ in the context of semiconductors [40]. These states profoundly impact the switching behavior, leakage current, and voltage tolerance of the device. An increase in interface defects leads to higher interface states, which cause electron scattering within energy bands, thereby reducing carrier mobility and adversely affecting device performance. Furthermore, the interface contact resistance or Schottky barrier at the metal-semiconductor junction is vital for the performance of new transistor devices, particularly those fabricated from two-dimensional (2D) materials or carbon-based compounds [41,42]. Challenges such as semiconductor-metal electrode contacts, band mismatches, and interface states can impede carrier transport, potentially causing energy level distortions, increased interface resistance, and ultimately, compromised device efficiency. Therefore, the investigation of device interfaces [43–46] remains a critical pathway towards enhancements of the functionality and performance of semiconductor devices. By understanding and mitigating interface-related issues such as trapped charge density and contact resistance, researchers can advance the development of more efficient and reliable electronic devices.

**Figure 4.**
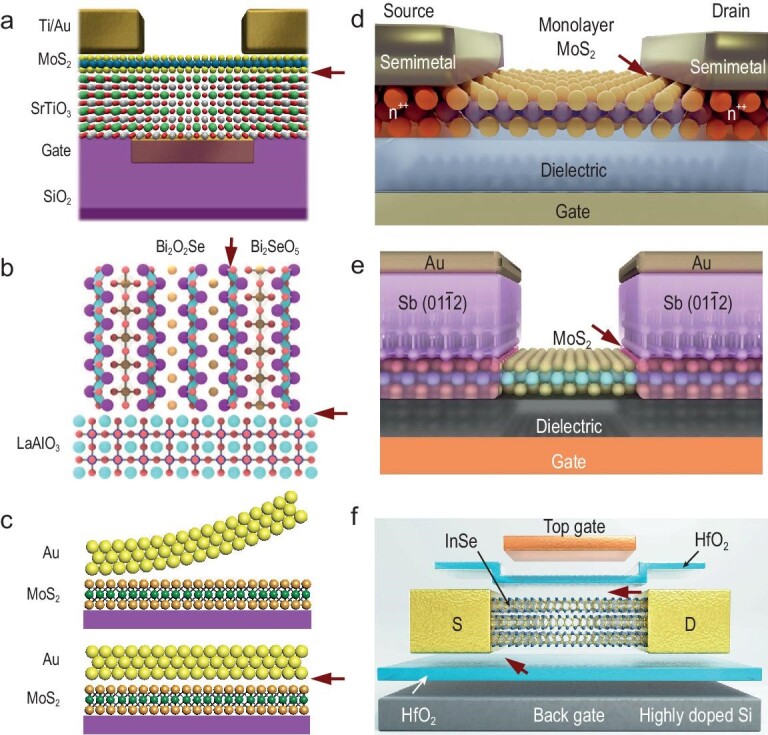
Several investigated interface systems in device, with interfaces marked by red arrows. (a) A 2D field-effect transistor featuring a SrTiO_3_ gate dielectric and MoS_2_ interface [51]. Copyright 2022, American Association for the Advancement of Science. (b) A 2D fin field-effect transistor designed with a Bi_2_O_2_Se base and its oxidized form Bi_2_SeO_5_ [52]. Copyright 2023, Springer Nature. (c) A technique for reducing interface defects in 2D materials through the application of an electrode layer transfer method [54]. Copyright 2018, Springer Nature. (d) Achievement of ohmic contact at the interface between semimetal Bi and MoS_2_ [55]. Copyright 2021, Springer Nature. (e) The contact resistance at the Sb(01$\bar{1}$2) and MoS_2_ interface nearing the theoretical quantum limit [56]. Copyright 2023, Springer Nature. (f) A transistor engineered from 2D InSe material, integrated with an HfO_2_ dielectric layer and yttrium-doped electrodes [57]. Copyright 2023, Springer Nature.

The interplay between semiconductors and oxides, particularly the Si and SiO_2_ interface, characterized by its relatively low interface state density, has been a driving force behind the adoption of silicon-based chips. The search for high-k gate dielectrics as alternatives to SiO_2_ emphasizes the need for low interface state densities [47]. Studies of other high-k gate materials [48–50], susceptible to oxide formation such as As_2_O_3_, Ga_2_O_3_, and HfO_2_, display interface state densities ranging from 10^11^ to 10^13^ cm^−2^ eV^−1^, significantly higher than the 10^10^ to 10^11^ cm^−2^ eV^−1^ observed at the Si/SiO_2_ interface. Research by Li *et al.* [51] into high-k single crystal perovskite strontium titanate films as gate dielectrics for 2D field-effect transistors (Fig. 4a) demonstrated that the van der Waals interface between strontium titanate and 2D semiconductors effectively lowers interface state densities. Innovative efforts by Peng *et al.* [52,53] on epitaxial high-k gate dielectric integrated 2D fin field-effect transistors (Fig. 4b), utilizing Bi_2_O_2_Se semiconductors and their oxide Bi_2_SeO_5_, exhibited exceptional performance metrics—mobility (270 cm^2^/Vs), off-state current (1 pA/μm), and a current on/off ratio of 10^8^ due to the excellent lattice compatibility between Bi_2_O_2_Se and Bi_2_SeO_5_.

Addressing metal-semiconductor contacts, Duan *et al.* [54] employed the electrode layer transfer method to reduce interface-induced defects from molecular beam epitaxial growth processes on 2D materials (Fig. 4c), bringing metal-semiconductor contact closer to the Schottky–Mott limit. Kong *et al.* [55] utilized semimetal Bi as a contact electrode for 2D materials, capitalizing on its minimal state density at the Fermi level to eliminate the Schottky barriers, thereby achieving ohmic contact (Fig. 4d). Wang *et al.* [56] made strides in enhancing orbital hybridization at the semimetal-2D semiconductor interface (Fig. 4e), resulting in reduced contact resistance for single-layer MoS_2_ to 42 Ω·μm, surpassing traditional silicon-based transistor contacts and nearing the theoretical quantum limit. Leveraging high-k ultra-thin dual-gate hafnium oxide (HfO_2_) dielectrics (Fig. 4f), along with yttrium-induced 2D phase transition strategies, Peng *et al.* [57] addressed semiconductor-metal contact challenges in 2D device applications, leading to high-performance InSe field-effect transistors with ohmic contacts, enhanced gate efficiencies, and ballistic rates approaching ideal conditions for ultra-short channels.

Another pivotal class of devices is sensors which are designed to convert external stimuli such as force, temperature, light, sound, and chemical composition into electrical signals. This concept traces back to 1829 with Nobili's invention of the thermocouple thermometer, based on the variation of the contact potential difference at the junction between two metals at different temperatures. The expansion of semiconductor technology has expedited the widespread adoption of semiconductor sensor devices, utilizing their intrinsic sensitivity to external conditions. Milestones in sensor development include introduction of the infrared sensor by Case in 1917, creation of the silicon diaphragm micromechanical pressure sensor by Peterson in 1954, the debut of the semiconductor metal oxide gas sensor in 1962, and the development of charge-coupled device (CCD) imaging sensor technology by Boyle and Smith [58] in 1970.

Despite the diverse range of sensor devices and their broad applications, their fundamental operation relies on interfaces and the electrical effects generated at these junctions [59]. Gas sensors, for example, function based on the charge alterations caused by the adsorption of gas molecules at the sensor interface, which leads to changes in the sensor voltage or current. CCD sensors utilize the storage and transfer of charges at interfaces for high-sensitivity and high-resolution imaging, while semiconductor sensors [60] often employ the unidirectional conductive properties of PN junction interfaces and the current amplification effects of transistor interfaces. Ongoing research into sensor devices has shown that interface control can significantly enhance sensor performance [61–63]. For instance, research by Peng *et al.* [64,65] demonstrated that sensors incorporating field-effect transistors exhibit increased sensitivity when the channel material at the interface is made thinner and more compact. The use of carbon nanotubes to create interfaces has enabled wafer-level batch production of sensors, achieving remarkable stability and uniformity. Fan *et al.* [66,67] have developed high-performance biosensors by leveraging functional biological interfaces with efficient biomolecular recognition and multivalent synergistic functions. Additionally, Chi *et al.* [68] have attained ultra-high sensitivity in NO_2_ detection through interface doping between self-assembled monolayers and perylene films, indicating that suitable deep trap states at the interface contribute to high sensitivity and ultra-fast response/recovery times.

These developments highlight the crucial role of interfaces in enhancing the performance of chip devices, which are essential for merging the material and digital worlds and foundational to achieving a highly interconnected, automated, and intelligent material domain. Deep understanding of the physicochemical properties and operational principles of functional interface systems is vital for creating sensors that excel in performance and withstand extreme conditions, offering limitless possibilities for the innovation of chip devices that go beyond Moore's Law and underscore the invaluable contribution of interface studies to future technological progress and innovation.

## THE SPECIFICITY OF INTERFACES

The pivotal role that interfaces serve in diverse fields such as superconductivity, catalysis, and device is fundamentally connected to their unique properties. Crystals, characterized by their periodicity and continuity, have their structural diversity constrained to seven crystal systems and fourteen lattice types. This limitation allows theoretical models to concentrate on the atoms contained within the unit cell. Utilizing the Born-Oppenheimer adiabatic approximation along with mean-field approximation simplifies the complex Schrödinger equation, which governs electron motion, to a more manageable form,


(1) \begin{eqnarray*}
{\boldsymbol{H\psi }}\left( {\boldsymbol{r}} \right) = \left[ { - \frac{{{{\hbar }^2}}}{{2m}}{{\nabla }^2} + {\boldsymbol{V}}\left( {\boldsymbol{r}} \right)} \right]{\boldsymbol{\psi }}\left( {\boldsymbol{r}} \right) = {\boldsymbol{\varepsilon \psi }}\left( {\boldsymbol{r}} \right),
\end{eqnarray*}

where ${\boldsymbol{H\ }}$symbolizes the Hamiltonian, which includes both the kinetic energy component $- \frac{{{{\hbar }^2}}}{{2m}}{{\nabla }^2}$ and the potential energy component ${\boldsymbol{V}}( {\boldsymbol{r}} )$, where ${\boldsymbol{\varepsilon }}$ denotes energy and ${\boldsymbol{\psi }}$ represents the electron wave function. In the context of crystals, the potential energy ${\boldsymbol{V}}( {\boldsymbol{r}} )$ is characterized by its periodicity, expressed as ${\boldsymbol{V}}( {\boldsymbol{r}} ) = {\boldsymbol{V}}( {{\boldsymbol{r}} + {{{\boldsymbol{R}}}_{\boldsymbol{n}}}} )$, where ${{{\boldsymbol{R}}}_{\boldsymbol{n}}}$ represents a periodic lattice vector. The square of the electron wave function, which reflects the electron probability density, provides insight into the electron state. Owing to the periodic potential of the crystal, Bloch's theorem asserts that the electron wave function assumes the Bloch form: ${\boldsymbol{\psi }}( {\boldsymbol{r}} ) = {{{\boldsymbol{e}}}^{ - {\boldsymbol{ikr}}}}{{{\boldsymbol{u}}}_{\boldsymbol{k}}}( {\boldsymbol{r}} )$, where ${\boldsymbol{k}}$ is the wave vector, and ${{{\boldsymbol{u}}}_{\boldsymbol{k}}}( {\boldsymbol{r}} )$ is a periodic function that echoes the crystal periodicity, fulfilling the condition ${{{\boldsymbol{u}}}_{\boldsymbol{k}}}( {{\boldsymbol{r}} + {\boldsymbol{R}}} ) = {{{\boldsymbol{u}}}_{\boldsymbol{k}}}( {\boldsymbol{r}} )$. Despite macroscopic solids consisting of an extensive number of atoms, their periodic structure facilitates the simplification of theoretical analysis to a limited number of atoms within a unit cell. The introduction of surfaces and interfaces interrupts this periodic structure, introducing the necessity for an additional surface/interface potential energy term in the Schrödinger equation. This adjustment is crucial to accurately describe the variations in potential energy that electrons experience in the vicinity of surfaces or interfaces:


(2) \begin{eqnarray*}
{\boldsymbol{H\psi }}\left( {\boldsymbol{r}} \right) &=& \left[ { - \frac{{{{\hbar }^2}}}{{2m}}{{\nabla }^2} + {\boldsymbol{V}}\left( {\boldsymbol{r}} \right) + {{{\boldsymbol{V}}}_{{Surf}/Interf}}\left( {\boldsymbol{r}} \right)} \right]\\
&& \times {\boldsymbol{\psi }}\left( {\boldsymbol{r}} \right) = {\boldsymbol{\varepsilon \psi }}\left( {\boldsymbol{r}} \right).
\end{eqnarray*}

The presence of aperiodic potentials undermines the effectiveness of the Bloch wave functions as comprehensive solutions. It becomes imperative to approximate the electron wave function as a combination of the Bloch states and aperiodic electron states (such as surface/interfacial states), wherein the Bloch states characterize the electron dynamics distant from the surface, and the surface/interfacial states are localized close to the surface or interface. The complexity and aperiodicity inherent in the surface/interface potential energy frequently make analytical solutions elusive. The theory developed by Tamm in 1932, which introduced a step potential barrier to model 1D semi-infinite crystal surface states, typically demonstrates an exponential decay localized near the surface, formally described as ${\boldsymbol{\psi }}( {\boldsymbol{x}} ) = {\boldsymbol{A}}{{{\boldsymbol{e}}}^{ - {\boldsymbol{Kx}}}}$, where ${\boldsymbol{K}}$ denotes the decay coefficient. The Tamm surface states exist independently from the crystal's bulk electron structure.

In 1939, Shockley [69] explored a 1D finite chain crystal, suggesting that surface states emerge from the dangling bonds of surface atoms, inherently linked to the bulk band structure of the crystal, thus adding a layer of complexity. Later efforts to solve for the surface states numerically have employed various models, including the nearly free electron model, the tight-binding model, and the self-consistent pseudopotential method, showcasing the complex nature of surface states. However, the importance of surface states has been consistently emphasized: In 1947, Bardeen highlighted the role of intrinsic surface states within the semiconductor bandgap, and the development of the first point-contact transistor was inspired by the observation of current amplification effects through the formation of a thin p-type surface layer on an n-type germanium semiconductor with gold electrodes; semiconductor surface states are known to cause Fermi level pinning, which is crucial for Schottky barrier formation; metal surface states are instrumental in creating 2D electron gases, essential for superconductivity; insulator surface states form the basis for topological insulators, facilitating quantum spin Hall and anomalous quantum Hall effects; surface light-induced electron resonance states, or surface plasmons, promote surface waveguide phenomena; and the role of surface states in molecular adsorption at surfaces is vital for catalytic activity.

Interfaces, created by the juxtaposition of two distinct materials or phases, introduce a significantly higher degree of complexity and a wider range of possibilities compared to monophasic surfaces, thereby amplifying the challenges inherent in their study. The diversity of interfaces is vast, and the blending of different substances can generate an endless variety of interfaces. Even interfaces formed by materials with identical chemical compositions can exhibit a wide array of structural diversities due to variations in the angles between crystal phases and lattice mismatches. This structural heterogeneity of interfaces offers a unique platform for tunability, presenting opportunities to profoundly alter the physical and chemical properties of materials. These alterations can lead to the emergence of new properties or modify existing ones, driven by complex underlying microscopic mechanisms that include interfacial electronics, electromagnetic interactions, and mechanical forces [70].

### ‘Creating the new from nothing’— interface-generated physical properties

**Figure 5.**
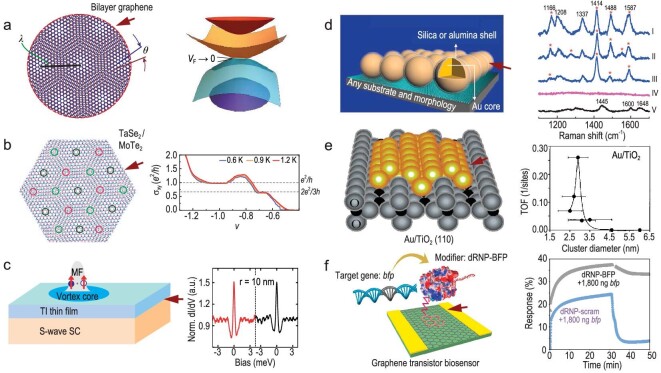
Some functional interface systems and their distinctive characteristics. The interfaces are marked by red arrows. (a) Magic-angle graphene showcasing flat band superconductivity [71]. Copyright 2018, Springer Nature. This interface features a unique arrangement of graphene layers at a specific twist angle, leading to the emergence of flat energy bands, which facilitates superconductivity at higher temperatures compared to conventional materials. (b) The interface between TaSe_2_ and twisted MoTe_2_, manifesting fractional quantum anomalous Hall effects [76]. Copyright 2023, American Physical Society. In this system, the interface between two transition metal dichalcogenides exhibits exotic quantum phenomena known as fractional quantum anomalous Hall effects, offering potential applications in topological quantum computing. (c) The interface between the superconductor NbSe_2_ and the topological insulator Bi_2_Te_3_, revealing Majorana zero mode [77]. Copyright 2016, American Physical Society. At this interface, the combination of a superconductor and a topological insulator gives rise to Majorana zero modes, which are promising for fault-tolerant quantum computing due to their non-Abelian statistics. (d) Nanoparticle interfaces known for amplifying molecular Raman signals [83]. Copyright 2010, Springer Nature. These interfaces involve nanoparticles that enhance Raman scattering signals from molecules adsorbed on their surfaces, enabling sensitive molecular detection in various fields such as biology and chemistry. (e) The interface between Au and TiO_2_, demonstrating improved CO oxidation activity [25,84]. Copyright 1998, American Association for the Advancement of Science and Copyright 2006, American Chemical Society. This interface exhibits enhanced catalytic activity for the oxidation of CO, offering potential applications in environmental remediation and renewable energy technologies. (f) Graphene transistor biosensor interface, augmented with functional groups to boost sensing signals [61]. Copyright 2019, Springer Nature.

Interfaces, the regions where two distinct materials or phases meet, can manifest novel physical properties not present in the constituent materials or phases themselves. In specific heterostructures, such as those involving 2D materials, novel properties like superconductivity can manifest at the interface, despite the interfacing materials individually lacking these characteristics. For example, graphene, known as a semimetal, lacks independent superconducting characteristics. However, Jarillo-Herrero *et al.* [71,72] experimentally discovered that aligning two graphene layers at a precise rotational angle, termed the ‘magic angle,’ creates a unique flat band structure at the interface, granting superconducting properties to the graphene interface (Fig. 5a). Similarly, the coupling in oxide heterostructures can lead to new magnetic states at the interface. Interfaces between two antiferromagnetic oxides have shown ferromagnetism [73,74], while antiferromagnetic order has been induced at the interface between diamagnetic copper-based superconductors and ferromagnetic manganese oxide [75].

Furthermore, interfaces can be the birthplace of novel quantum phenomena. Li *et al.* [76] engineered an interface between the 2D layered metal TaSe_2_ and twisted MoTe_2_. Upon forming an ohmic contact, this interface system displayed a fractional quantum anomalous Hall effect, a phenomenon not seen in individual materials (Fig. 5b). Jia *et al.* [77] observed Majorana zero modes at the interface by epitaxial growth of high-quality topological insulator thin films of Bi_2_Te_3_ on NbSe_2_, marking a pivotal advancement in the exploration and manipulation of the physical properties of topological insulators (Fig. 5c). Additionally, in 2006, Zhang [78] proposed the realization of the quantum spin Hall effect at the HgTe-CdTe quantum well interface, a prediction experimentally verified in 2007 [79].

### ‘Enhancing the weak to strong’— interface-augmented physical properties

The distinctive properties of interfaces can remarkably amplify the efficacy of specific physical or chemical phenomena. For example, Xue *et al.* [11] have demonstrated interface-enhanced superconductivity at the boundary between FeSe and SrTiO_3_, where a superconducting gap of ∼20 meV and a transition temperature reaching 65 K were measured, greatly surpassing the inherent superconducting temperature of FeSe. This finding holds significant promise for the advancement of high-temperature superconducting materials. In explorations of materials science, the impact of interface effects can lead to considerable alterations in their piezoelectrics [80], ferroelectrics [81], and electrostriction [82].

In nanoscience, interfaces between nanoparticles can generate electron resonance states, known as plasmons, which can both initiate waveguide properties and substantially amplify Raman signals, a phenomenon referred to as interface-enhanced Raman scattering. Tian *et al.* [83] harnessed this effect to capture Raman spectra of various molecules or ions attached to platinum and gold single-crystal electrodes under electrochemical conditions, employing shell-isolated nanoparticles (Fig. 5c). Moreover, interfaces can significantly boost catalytic efficiency, as discovered by Goodman *et al.* [25,84] who found that charge transfer at the Au-TiO_2_ interface leads to a negatively charged Au surface, greatly enhancing the Au catalytic capability for CO oxidation (Fig. 5c). In photocatalysis, the design of specific interface structures can improve the separation efficiency of photogenerated carriers, thus elevating the performance of photocatalytic reactions [85]. In electrocatalysis, fine-tuning the interface structure of electrode materials can profoundly enhance electrocatalytic reaction activity. Interface modification and control have also proven to significantly intensify sensing signals, as exemplified by Aran *et al.* [61] who enhanced a graphene transistor sensor interface with CRISPR-Cas9 genes, significantly boosting the biosensing signal for swift and selective identification of specific sequences within complete genome DNA (Fig. 5d).

## THE KEY TO INTERFACIAL SCIENCE— INTERFACIAL STATES

**Figure 6.**
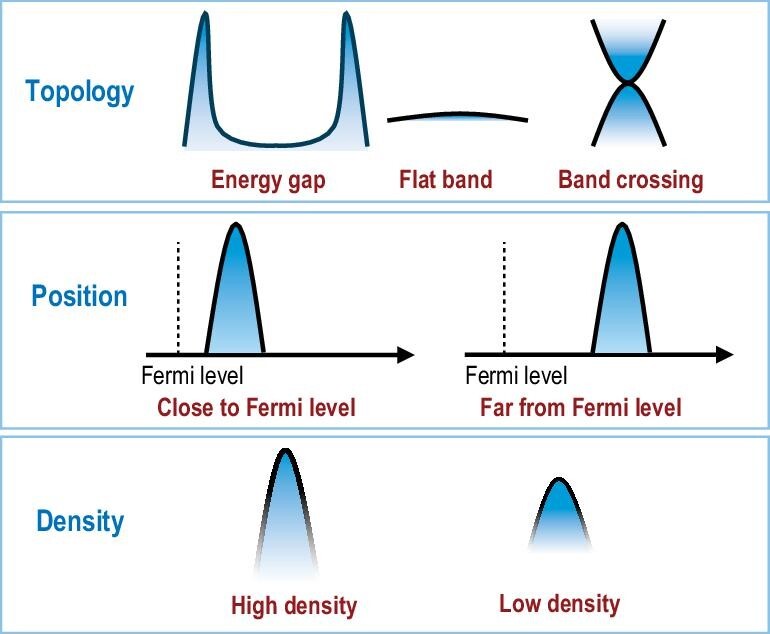
Basic properties of interfacial states: topology, position, and density.

In the development of surface science, the concept of surface states has become a pivotal element connecting the microstructural aspects of materials with their macroscopic properties, serving as fundamental topics and guiding principles for the swift progress of that field. Similarly, it is anticipated that interfacial electronic states will occupy a central role in the intricate domain of interfacial science research. The distinct characteristics of interfaces are often a result of their unique electronic states, which are influenced by the interfacial structure and composition. While bearing resemblance to surface states, interfacial electronic states [86], termed ‘interfacial states’, differ from the same term used in information and device-related contexts. These electronic states at interfaces are characterized by specific features such as their topology (for instance, energy gap, flat band, and band crossing), their spatial position relative to the Fermi level, and their density, which can vary from high to low (Fig. 6).

### Topology of interfacial states

The occurrence of superconducting gaps or flat bands in proximity to the Fermi level marks the essence of interface superconductivity. The relationship between the gap width ($\Delta $) and the transition temperature (${{T}_c}$) is linear in the BCS theory [3], defined by


(3) \begin{eqnarray*}
{{T}_c} = \Delta /1.764{{k}_{B,}}
\end{eqnarray*}

where$\ {{k}_B}$ is the Boltzmann constant [87]. The design and manipulation of interfaces to foster states with superconducting gaps or flat bands near the Fermi level are crucial for developing superconducting systems at interfaces. Additionally, the appearance of crossing shapes in interfacial states suggests quantum topological phenomena [88–90], as evidenced by the discovery of the quantum spin Hall state at the CdTe and HgTe interface [79].

### Position of interfacial states

The d-band center theory, proposed by Nørskov [91–93], indicates a relationship between the position of the d-band center in catalyst metals and their adsorption energy difference:


(4) \begin{eqnarray*}
{\mathrm{\Delta }}E = \pi {{V}^2}\Sigma \delta \left( {{{\varepsilon }_i} - {{\varepsilon }_d}} \right),
\end{eqnarray*}

where *V* is the coupling matrix elements and ${{\varepsilon }_i} - {{\varepsilon }_d}$ is the energy difference from the *i* state to the d-band center [93]. Therefore, the position of the d-band center significantly affects the molecular adsorption energy. According to the Sabatier principle, a volcano-like correlation exists between the catalytic activity of a metal and its adsorption energy, highlighting the significance of the d-band center's position in determining catalytic efficacy. For instance, the relatively low reactivity of bulk Au is linked to its d-band center's considerable distance from the Fermi level, but adjusting the position of Au's d-band center can notably improve its catalytic activity.

### Density of interfacial states

The density of interfacial trapped charges, also known as interface states within the semiconductor field, significantly influences various aspects of transistor devices, including switching behavior, leakage current, field-effect mobility, and threshold voltage [94]. The shift of the threshold voltage $\Delta {{V}_{th}}$ with respect to its intrinsic position depends on the value of interface states and the geometrical capacitance of the interface ${{C}_i}$ [95]:


(5) \begin{eqnarray*}
{{V}_{th}} = - \frac{{\mathop \smallint \nolimits_{Ei}^{Ef} e{{D}_{it}}dE}}{{{{C}_i}}}.
\end{eqnarray*}

At the metal-semiconductor interface, the occurrence of the Fermi level pinning, represented by the pinning factor *S*, is influenced by the density of interface states ${{D}_{it}}$ as $S = 1/( {1 + {{e}^2}{{D}_{it}}\delta /{{\varepsilon }_0}} )$, where $\delta $ is the width of the interfacial dipole layer [96,97]. The pinning factor $S = 0$ indicates that the Fermi level of the interface is strongly pinned by the interface states. Therefore, adjusting the density of interfacial states is pivotal for enhancing the performance of semiconductor devices.

In summary, the fundamental characteristics of interfacial states profoundly dictate the functionalities and properties of interfaces: the configuration of these states is pivotal for engendering interface superconductivity and setting the superconducting temperature; the spatial positioning of interfacial states plays a crucial role in influencing the catalytic efficiency of interface systems; and the concentration of interfacial states is directly tied to the operational efficacy of devices at interfaces. Hence, establishing a quantitative relationship between the electronic states at interfaces and their functional outcomes represents a significant scientific endeavor within the realm of interfacial science. Achieving this correlation is essential for elevating superconducting temperatures at interfaces, enhancing the catalytic performance of interfaces, and advancing the functionality of devices leveraging interface phenomena.

## PERSPECTIVE OF INTERFACIAL SCIENCE

### Significant challenges in interfacial science

The significance and distinctiveness of interfaces are well acknowledged within the science community. Nevertheless, the study of interfacial science is confronted with several formidable challenges due to the heterogeneous, non-periodic, and discontinuous characteristics of interfaces.

#### Invisibility in interface detection

Interface detection is essential in interfacial science, but faces significant hurdles. Interfaces are often concealed within solid materials, making them difficult to be accessed by the signal probes of the employed detection techniques, thus limiting the effectiveness of conventional surface characterization methods for direct interface detection. In addition, the signals emanating from interfaces, which are essential for detection, struggle to penetrate the solids to reach their detectors. Complication further arises due to the low abundance of interface atoms and the subtlety of their signals, making it tremendously challenging to determine whether these signals are emanating from the interface or the bulk material, inhibiting the application of traditional solid state characterization techniques for interface analyses. These obstacles collectively impede the thorough investigation and comprehension of interface structures and the mechanisms behind the formation of interfacial states.

#### Incalculability in interface computation

Achieving an accurate theoretical description and simulation of the intrinsic properties of interfaces is extremely challenging. The intersection of two or more materials at interfaces results in complex and variable structures. Interface phenomena driven by thermodynamics, such as mutual diffusion, reaction rearrangement, defect formation, lattice mismatches, and charge and energy transfers, often interplay in complex ways. These descriptions must incorporate spatial and temporal changes occurring at the interface, and account for interactions ranging from weak van der Waals forces to strong chemical bonds. This necessitates a nuanced approach to capturing the distance-dependence, directionality, and time-dependence of these multiscale interactions, thereby complicating theoretical calculations, and making them labor-intensive.

#### Imperfection in interface construction

For practical applications, highly ordered structures often offer superior performance and specific functionalities that are more readily controlled. The customization of interfaces requires controlled and precise construction techniques. However, according to the second law of thermodynamics, materials within isolated systems tend towards increased entropy, which can lead to complexes such as interface component diffusion, lattice mismatches, interface defects, and reaction rearrangement. These factors pose challenges to the precise fabrication of highly ordered interfaces.

All these challenges underscore the intricate nature of interfacial science and highlight the necessity for innovative strategies in detecting, modeling, and constructing interfaces with high precision. Such advancements will pave the way for significant progress in interfacial science and its practical applications.

### Future directions of interfacial science

**Figure 7.**
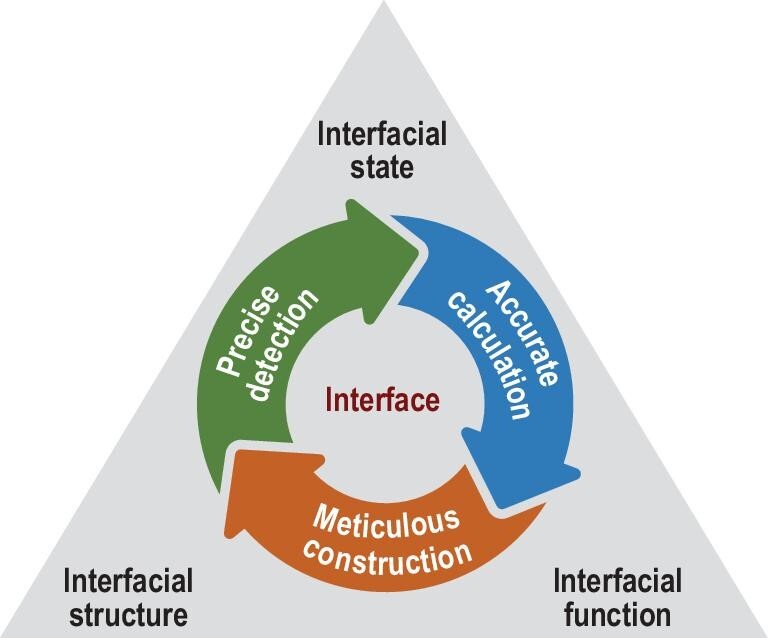
Diagram illustrating the development directions in interfacial science: precise detection, accurate calculation, and meticulous construction of interfaces.

The structure and function of interfaces are the foundation of interfacial science. Interfacial states serve as the bridge connecting the structures and functions of interfaces. Advancements in interfacial science should pivot around precise detection, accurate calculation, and meticulous construction of the interfaces. This necessitates the creation of novel interface detection tools and fabrication techniques, alongside establishing a fresh theoretical foundation for interfaces, thus steering the holistic progression of future technologies reliant on functional interface systems (Fig. 7).

#### Precise detection of interfaces

To surmount the challenge of ‘invisibility’ in interface detection, a feasible approach involves redefining interface detection as surface detection. This could be accomplished through methods such as dimensional reduction or longitudinal sectioning, thereby rendering obscured interfaces more amenable to study using existing surface science methodologies [98,99]. Additionally, pioneering new physical concepts and technical approaches, like employing probe signals capable of deep penetration like positron annihilation and neutron detection techniques, could bridge the gap in detecting interfacial states and allow for direct observation of electronic states deep inside solids. Moreover, developing methodologies to dissect and amplify faint interface signals could facilitate the precise differentiation and analysis of signals emanating from interface and bulk electronic states, thus enabling accurate identification of interfacial states. Further developments of *in-situ* or *operando* high-resolution detection techniques can also help monitor interface dynamics under operational conditions, which would provide valuable insights into the evolution of interfacial properties.

#### Accurate calculation of interfaces

To address the ‘inaccuracy’ issue in interface characterization, it is imperative to refine and expand theoretical models that can accurately describe the complex dynamics at interfaces. Efforts should focus on calculating the attributes of non-periodic, discontinuous, and heterogeneous interface systems, devising computation and simulation strategies for accurately depicting diffusion and reaction dynamics, defect generation, lattice mismatches at interfaces, and processes of charge and energy transfer. Enhancing algorithm efficiency is also crucial for optimizing computational resources dedicated to the intricate modeling of interfacial states. In addition, artificial-intelligence–based methods can offer new possibilities that enhance searching efficiency in discovering and understanding complex interface structures and properties.

#### Meticulous construction of interfaces

To tackle the ‘imperfection’ issue in interface fabrication, developing atomic-level precision construction methodologies is essential. Such techniques should enable exact manipulation of interfacial structure and electronic states, informed by insights into the mechanisms governing changes in interfacial states. Understanding how interfacial states influence the properties of interface systems could lead to precise forecasting of interface structural effects on properties and functionalities. A systematic approach to engineering interfacial states would permit fine-tuning of these states for targeted functional control.

These strategic directions emphasize the importance of pushing the boundaries of interfacial science through theoretical breakthroughs, technological innovations, and their applications in practice. This concerted effort is set to catalyze significant advancements in physical science, electronics, and catalysis, heralding a new era of technological evolution.

## SUMMARY

The emergence and progress of surface science have catalyzed a range of significant scientific and technological milestones, directly and indirectly contributing to industrial advancements. A prime example is IBM's launch of cutting-edge 2-nanometer chip technology in 2021, followed by the introduction of a quantum computer with a groundbreaking qubit count in 2022. These achievements have their roots in the company's fundamental research into insulator surface silicon growth initiated in 1982 [100] and Josephson junction surface loss explored in 1986 [101]. Similarly, surface science research has played a crucial role in industrial transformations, such as the development of coal-to-oil technology by Synfuels China Technology Co., Ltd. In partnership with Shenhua Group, they built the world's largest coal-to-oil plant in 2016, boasting an annual capacity of 4 million tons, based on their research on iron-based Fischer–Tropsch synthesis begun in 1995 [102]. These examples underscore that those major technological innovations are deeply intertwined with the accumulation of extensive fundamental scientific research.

By advancing precise detection tools, accurate computational methods, and meticulous construction techniques for interfaces, and by laying down a robust research and theoretical framework for understanding interfacial states, pivotal breakthroughs in fundamental research are within reach. This progress is bound to robustly propel the evolution of future technologies including but not limited to the following aspects: elevating interface superconducting temperatures for electrical transmission applications; broadening interface superconducting material systems for maglev train technologies; pioneering interface quantum topological systems for topological quantum computing; optimizing catalytic processes like Fischer–Tropsch synthesis to reduce energy consumption, carbon emissions, and precious metal reliance, thus advancing new energy catalysis; enabling efficient and swift intelligent sensing for the Internet of Things; creating multifunctional portable sensors for real-time health monitoring; and improving weak signal detection capabilities to even support the search for extraterrestrial life. The potential and prospects for future technologies based on interfacial science are both vast and promising.
